# Epidemiology of sepsis in Beijing from 2012 to 2018: analysis of hospital homepage databases derived from the Beijing Public Health System

**DOI:** 10.1186/s12889-022-14725-1

**Published:** 2022-11-30

**Authors:** Dong-chen Guo, Hai-zhou Zhuang, Jin Lin, De-yuan Zhi, Mei-li Duan

**Affiliations:** Department of Critical Care Medicine, Beijing Friendship Hospital, Capital Medical University, No. 95 Yong’an Road, Xicheng District, 100050 Beijing, China

**Keywords:** Sepsis, Epidemiology, Mortality, Cost

## Abstract

**Background:**

We aimed to evaluate the epidemiology of sepsis in secondary and tertiary hospitals in Beijing, China between 2012 and 2018 using information derived from the Beijing Public Health System.

**Methods:**

The Beijing Public Health System accessed hospital homepage databases and identify patients who diagnosed sepsis or associated condition according to the International Classification of Diseases, 10th Edition, Clinical Modification codes. There are 125 hospitals involved in this study, including 61 secondary hospitals, accounting for 49.2%, and 63 tertiary hospitals, accounting for 50.8%. Patients were stratified by age as minors (0–17 years old), adults (18–64 years old), seniors (65–84 years old), and the elderly (≥ 85 years old). Patient’s demographic information, treatments, outcomes, and all-cause hospitalization cost were evaluated.

**Results:**

This study involved 8,597 patients. Patients treated in tertiary hospitals or received blood transfusion decreased with age, while patients who were male, received ventilation, or took Traditional Chinese Medicine, and in-hospital mortality and hospitalization cost, increased with age. There were 2,729 (31.7%) deaths in this study. A slight increase in in-hospital mortality occurred from 2012 to 2018. Median hospitalization cost for all patients was ¥29,453 (15,011, 65,237). Hospitalization cost showed no significant change from 2012 to 2016, but increased in 2017 and 2018.

**Conclusion:**

Sepsis is associated with high mortality and cost. From 2012 to 2018, in-hospital mortality and hospitalization cost of sepsis in Beijing increased significantly with age, and slightly by year.

## Background

Sepsis is associated with high morbidity and mortality. Worldwide, sepsis represents a major health concern in patients in intensive care units (ICU). Notably, the incidence of sepsis has risen in the last decades [1–3], causing a substantial clinical and economic burden to healthcare systems [4, 5]. Globally, in 2012, 29.5% of patients admitted to the ICU experienced sepsis and ICU mortality rates in patients with sepsis were 29.8% [6, 7], while hospital mortality rates were 35.3% [6]. In 2017, the Global Burden of Diseases, Injuries and Risk Factors Study reported 49 million cases of sepsis and 11 million sepsis-related deaths, which accounted for approximately 19.7% of all deaths worldwide [8]. In 2015, the standardized sepsis-related mortality rate in China was 66.7 deaths per 100,000 population, resulting in an estimated 1 million sepsis-related deaths [9]. Long-term epidemiological investigations on sepsis in China are limited. The objective of this study was to evaluate the epidemiology of sepsis in secondary and tertiary hospitals in Beijing between 2012 and 2018 using information derived from the Beijing Public Health System. Comparisons of the epidemiological characteristics, hospitalization and prognosis of sepsis among minors, adults and the elderly will help hospitals provide medical services that optimize length of stay, patient outcomes, and cost of hospitalization.

## Methods

### Ethics declarations

This study was approved by Bioethics Committee of our hospital and The informed consent was waived by Bioethics Committee of Beijing Friendship hospital. (No: 2021-P2-121-01).

### Data source

The Beijing Public Health System was used to access hospital homepage databases and identify patients admitted to secondary and tertiary hospitals in Beijing between 2012 and 2018 who had a diagnosis of sepsis or associated condition according to a primary International Classification of Diseases, 10th Edition, Clinical Modification (ICD-10-CM) code (Table 1). There are 125 hospitals involved in this study, including 61 secondary hospitals, accounting for 49.2%, and 63 tertiary hospitals, accounting for 50.8%. Patients admitted to military and private hospitals were excluded.

**Table 1:** Primary ICD-10-CM code

Diagnosis	ICD-10-CM
Sepsis	A41.902
Septic shock	A41.903
Acute renal injury	N17.901
Acute renal failure	N17.903
Infectious multiple organ dysfunction syndrome	R65.101

*ICD-10-CM* International Classification of Diseases, 10th Edition, Clinical Modification

Patient selection was refined using secondary ICD-10-CM codes that aligned with the Third International Consensus Definitions for Sepsis and Septic Shock (Sepsis-3) recommendation that sepsis be defined as a life-threatening organ dysfunction caused by a dysregulated host response to infection [10] (Table 2).

**Table 2:** Secondary ICD-10-CM code

Diagnosis	ICD-10-CM
Bacteremia and fungemia	
Salmonella typhi, Salmonella typhimurium, Listeria, Streptococcus group A, Streptococcus pneumoniae, Streptococcus, Staphylococcus aureus, Staphylococcus epidermidis, Gram-negative bacterium, Escherichia coli, Proteusbacillus vulgaris, Gram-negative bacillus, Klebsiella, Pseudomonas Aeruginosa, Enterobacter cloacae, Acinetobacter, Enterococcus, Gram-positive bacteria, Candida, Fungus	A01.002, A02.102, A32.701, A40.001, A40.301, A40.901, A41.001, A41.102, A41.501, A41.511, A41.581, A41.583, A41.584, A41.586, A41.588, A41.589, A41.801, A41.803, B37.701, B49xx19
Septicemia	A41.901
Sepsis	A41.902
Septic shock	A41.903
Infectious shock	A41.908
Toxic shock syndrome	A48.301
Bacteremia	A49.901
Shock caused by pneumonia	J18.902
Toxic shock from infection	R57.801
Toxic shock	R57.802
Endotoxic shock	R57.803
Infectious multiple organ dysfunction syndrome	R65.101
Multiple organ dysfunction syndrome	R65.301
Multiple organ failure	R68.801
Systemic inflammatory response syndrome	R65.901
One of the main admission or discharge diagnoses is shock (R57.901) and the other is infectious disease	

*ICD-10-CM* International Classification of Diseases, 10th Edition, Clinical Modification.

### Data screening and database establishment

**Fig. 1:**
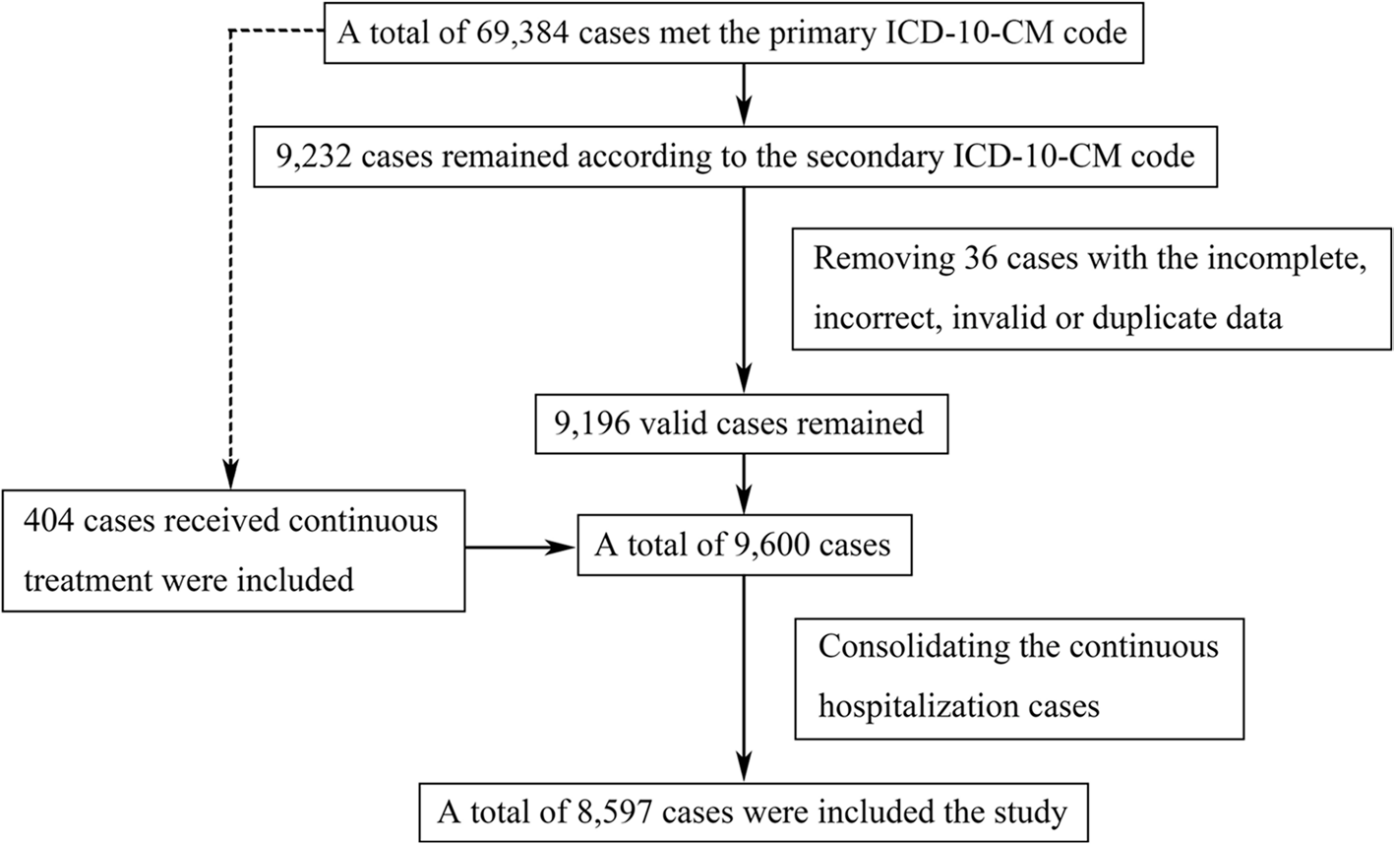
Flowchart of patient selection. ICD-10-CM: International Classification of Diseases, 10th Edition, Clinical Modification

A total of 69,384 patients with a diagnosis of sepsis or associated condition according to a primary ICD-10-CM code were identified, 9,232 patients had a secondary ICD-10-CM code, and 36 patients with incomplete, incorrect, invalid or duplicate data were excluded. Of the 69,384 patients with a diagnosis of sepsis or associated condition according to a primary ICD-10-CM code, 404 patients were discharged and readmitted to the same hospital within a few days, once or on multiple occasions, during which they had a secondary ICD-10-CM code. For the purpose of this study, these patients were considered to have received continuous treatment. After consolidating the data for these patients into a single hospitalization, the final dataset included information for 8,597 patients (Fig. 1).

### Data collection

Extracted data included patient’s demographic information (gender, age, year of hospitalization, hospital level), treatments (blood transfusion, ventilation, Traditional Chinese Medicine [TCM]), outcomes, and all-cause hospitalization cost. For patients who received continuous treatment, demographic data were reported based on the last hospitalization, and variables such as length of hospital stay, treatment and costs were cumulative across hospitalizations. Patients were stratified by age as minors (0-17 years old), adults (18-64 years old), seniors (65-84 years old) and the elderly (≥85 years old).

### Statistical analysis

Statistical analysis was performed using SPSS v25.0 (IBM, America), Categorical variables are reported as frequency (n) and percentage (%), and were compared with the Chi-square test [11]. Normally distributed continuous variables are reported as mean ± standard deviation, and were compared with the *T*-test or variance analysis. Abnormally distributed continuous variables are reported as median (25, 75 percentiles) [M (Q_L_, Q_U_)], and were compared with the Wilcoxon/Kruskal-Wallis nonparametric test. Temporal trends are presented as line graphs. *p*<0.05 was considered statistically significant.

## Results

### Demographic characteristics and general information

This study involved 8,597 patients, including 4,992 males (58.1%) and 3,605 females (41.9%) with a median age of 60 (50, 81) years. 7,617 (88.6%) patients were admitted to tertiary hospitals, 2,994 (34.8%) patients received blood transfusion, 2,286 (26.6%) patients received ventilation for a median 117 (33, 282) hours, and 4,677 (54.4%) patients were treated with TCM. Median length of hospital stay was 11 (6, 18) days. 2,729 (31.7%) patients died in hospital.

### Clinical characteristics of age groups

Patients were stratified by age as minors (0-17 years old; *n*=935, 10.9%), adults (18-64 years old; *n*=2,848, 33.1%), seniors (65-84 years old; *n*=3,526, 41.0%) and the elderly (≥85 years old; *n*=1,288, 15.0%). The demographic and clinical characteristics of patients stratified by age are summarized in Table 3. Except for platelet volume received during blood transfusion, there were significant differences in the demographic and clinical characteristics of all patients across age categories, and these variables showed a significant linear relationship with age. Specifically, the proportion of patients treated in tertiary hospitals or who received blood transfusion decreased with age, while the proportion of patients who were male, received ventilation, or took TCM, and in-hospital mortality and hospitalization cost, increased with age.

**Table 3:** Demographic and clinical characteristics of included patients stratified by age

Clinical characteristics	Minor *n*=935 (10.9%)	Adult *N*=2,848 (33.1%)	Elderly *N*=3,526 (41.0%)	Oldest *N*=1,288 (15.0%)	*χ*^2^/*H* ^a^	*p*	*χ*^2 b^	*p*_*L*_	*R*	*p*_*R*_
Male, n (%)	577 (61.7)	1,728 (60.7)	1,926 (54.6)^c^	761 (59.1) ^c^	30.775	<0.001	10.551	0.001	0.035	0.001
Age, year (IQR)	1 (1, 4)	53 (39, 59)	77 (71, 81)	88 (86, 89)						
Tertiary hospital, n (%)	934 (99.9)^c^	2,680 (94.1)^c^	2,997 (85.0)^c^	1,006 (78.1)^c^	389.163	<0.001	384.901	<0.001	-0.212	<0.001
Blood transfusion, n (%)	526 (56.3)^c,d^	756 (26.5)^c,d^	1,238 (35.1)^c^	474 (36.8)^d^	277.579	<0.001	13.167	<0.001	-0.039	<0.001
Red blood cell, IU (IQR)	1 (1, 3.75)^c,d,e^	4 (2, 9)^c^	4 (2, 8)^d,f^	4 (2, 6)^e,f^	65.916	<0.001				
Plasma, ml (IQR)	400 (200, 800)^c,d,e^	800 (400, 1600)^c^	600 (400, 1200)^d^	600 (400, 1200)^e^	26.358	<0.001				
Platelet, IU (IQR)	1 (1, 3)	2 (1, 3.75)	2 (1, 3)	2 (1, 3)	4.789	0.188				
Ventilation, n (%)	136 (14.5)^c^	534 (18.8)^c^	1,143 (32.4)^c^	473 (36.7)^c^	288.245	<0.001	263.938	<0.001	0.175	<0.001
ventilation time, h (IQR)	89.5 (24, 200)^c,d^	79 (20, 215.5)^e,f^	122 (39, 301)^c,e^	153 (46, 356.5)^d,f^	48.085	<0.001				
Chinese medicine, n (%)	366 (39.1)^c,d^	1,441(50.6)^c,d^	2,076 (58.9)^c^	794 (61.6)^d^	160.079	<0.001	146.911	<0.001	0.131	<0.001
Total cost, CNY (IQR)	16,780 (9,928, 36,678)^c,d^	23,540 (13,770, 47,538)^c,d^	36,934 (18,148, 78,793)^c^	41,215 (18,991, 88,259) ^d^	478.803	<0.001				
Outcome										
Hospital mortality, n (%)	45 (4.8)^c^	496 (17.4)^c^	1,440 (40.8)^c^	748 (58.1)^c^	1,129.596	<0.001	1,109.469	<0.001	0.359	<0.001
Hospital stay, d (IQR)	8 (6, 14)^c,d^	11 (7, 16) ^c,e^	12 (6, 19) ^c,e^	11 (5, 20) ^d^	57.719	<0.001				

*IQR* Interquartile range

*CNY* Chinese Yuan

^a^Chi-square or Kruskal-Wallis

^b^Linear-by-linear association

^c-f^The difference in pairwise comparison was statistically significant

Pairwise comparisons showed significant differences in gender between seniors and the elderly; hospital grade, mechanical ventilation and in-hospital mortality between all age groups; blood transfusion and the use of TCM between minors and adults; red blood cell volume received during blood transfusion between minors and the other age groups and adults and the elderly; and plasma volume received during blood transfusion between minors and the other age groups. There were significant differences between all age groups in ventilation time, except between minors and adults and seniors and the elderly; length of hospital stay, except between adults and seniors and seniors and the elderly; and hospitalization cost, except between seniors and the elderly.

### Comparison of in-hospital mortality across age groups

**Fig. 2:**
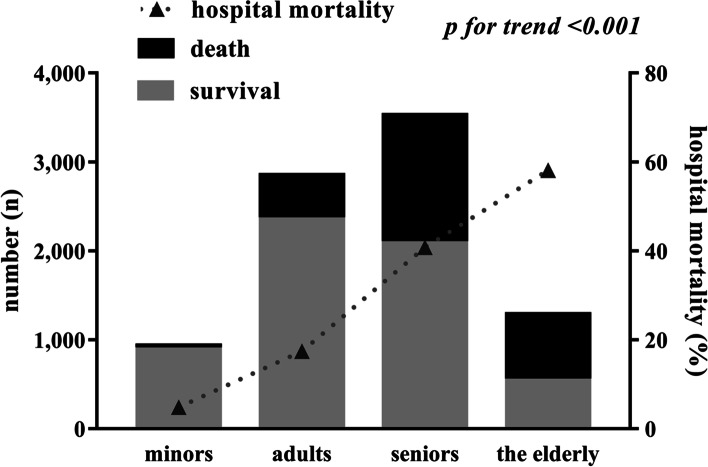
In-hospital mortality stratified by age

There were 2,729 (31.7%) in-hospital deaths from sepsis in this study. There was a significant difference in in-hospital mortality across hospital type, with 594 deaths (60.6%) occurring in secondary hospitals, and 2,135 deaths (28.0%) occurring in tertiary hospitals (χ^2^=425.443, *p*<0.001). In-hospital mortality was 4.8% (45/935), 17.4% (496/2,848), 40.8% (1,440/3,526), and 58.1% (748/1,288) for minors, adults, seniors and the elderly, respectively. There were significant differences in in-hospital mortality across age categories (*χ*^2^=1,129.596, *p*<0.001), and in-hospital mortality showed a significant linear relationship with age (*χ*^2^=1,109.469, *p*<0.001; *R*=0.359, *p*<0.001), indicating in-hospital mortality increased with age (Table 3, Fig. 2).

### Trends in in-hospital mortality from 2012 to 2018

**Fig. 3:**
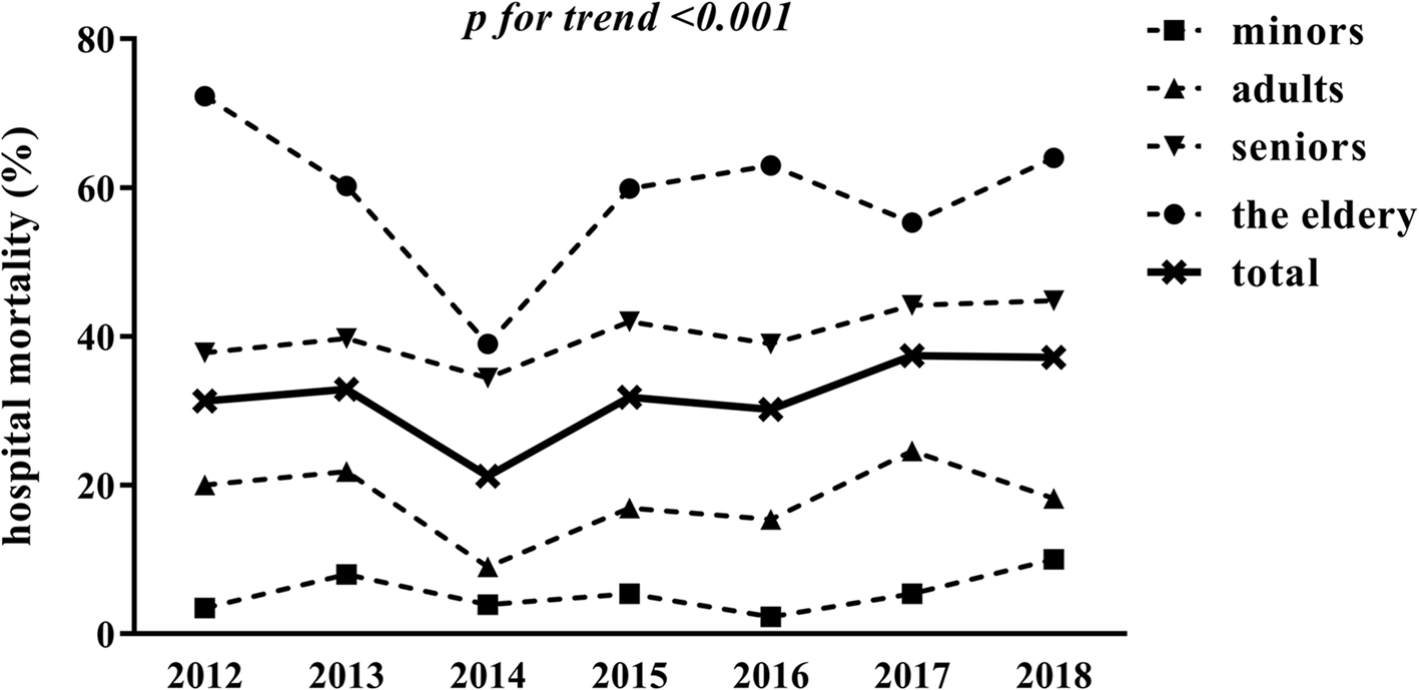
Trends in in-hospital mortality stratified by age from 2012 to 2018

In-hospital mortality was 31.3% (115/367), 32.9% (197/598), 21.2% (255/1204), 31.8% (553/1741), 30.2% (597/1975), 37.4% (725/1941) and 37.2% (287/771) for each consecutive year from 2012 through 2018, respectively. There were significant differences in in-hospital mortality across the years (*χ*^2^=103.401, *p*<0.001) and in-hospital mortality showed a significant linear relationship with time (*χ*^2^=40.909, *p*<0.001; *R*=0.069, *p*<0.001), indicating in-hospital mortality increased by year (Fig. 3).

### Trends in hospitalization costs across age groups from 2012 to 2018

**Fig. 4:**
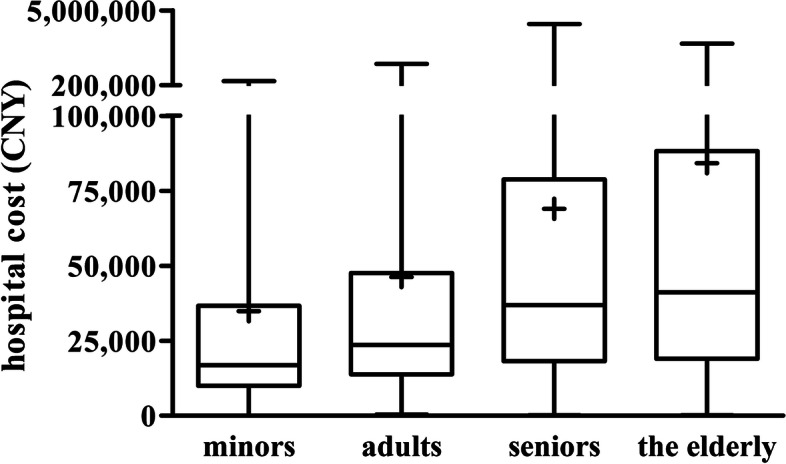
Box-plot of hospitalization cost stratified by age. CNY: Chinese Yuan

**Fig. 5:**
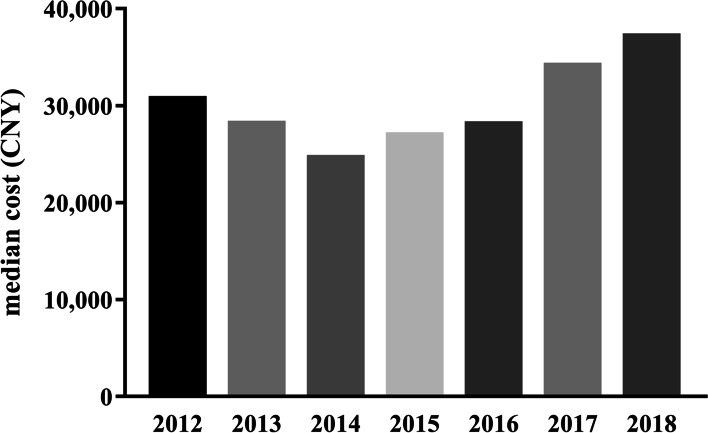
Median hospitalization cost of all patients by year from 2012 to 2018. CNY: Chinese Yuan

Median hospitalization cost for all patients included in this study was ¥29,453 (15,011, 65,237). There was no significant difference in median hospitalization cost across hospital type (secondary hospital, ¥31942.9 [14052.2, 69776.2]; tertiary hospital, ¥29250.3 [15072.6, 64939.8]; *p*<0.944). There were significant differences in median hospitalization cost for minors and adults, but not for seniors and the elderly (*H*=478.803, *p*<0.001) (Table 3, Fig. 4). There were significant differences in median hospitalization cost for all patients across the years (H=68.905, *P*<0.001). Pairwise comparison showed significant differences in median hospitalization cost for all patients between 2014 and 2017, 2014 and 2018, 2015 and 2017, 2015 and 2018, 2016 and 2017, and 2016 and 2018 (*p*<0.001). There was a numerical decrease in median hospitalization cost between 2012 and 2016, and a numerical increase between 2017 and 2018 (Table 3, Fig. 5).

## Discussion

This study used the Beijing Public Health System to access hospital homepage databases and to evaluate the epidemiology of sepsis in secondary and tertiary hospitals in Beijing between 2012 and 2018. The study included patients who had a diagnosis of sepsis or an associated condition according to ICD-10-CM codes. The median age of the study population was 60 (50, 81) years and 58.1 % were males. This age and gender distribution is similar to other studies investigating the epidemiology of sepsis in China [12]. Our findings showed that the proportion of patients with sepsis treated in tertiary hospitals or who received blood transfusion decreased with age, while the proportion of patients who were male, received ventilation or took TCM, and in-hospital mortality and hospitalization cost, increased with age.

The proportion of patients with sepsis treated in tertiary hospitals decreased with age from 99.9% of minors to 78.1% of the elderly. This may be related to different expectations of patients and their families around outcomes. Younger patients, especially minors, are expected to have a good prognosis. The prognosis for elderly patients may not be so good, so patients and families may choose the less invasive treatments provided by secondary hospitals, or palliative care.

A meta-analysis indicated that TCM combined with conventional treatment can improve the prognosis of patients with sepsis [13–15]. Statistics show the number of hospital beds in Beijing for patients is 6.85 per 1,000 population [16], while the number of hospital beds for patients treated with TCM is 10.48 per 10,000 population [17], accounting for only one sixth of the total. In this study, 54.4% of patients received TCM; thus, it can be inferred that many physicians in hospitals providing Western medical services also used TCM, and a combination of TCM and Western medicine was commonly used when treating sepsis.

A previous study showed the 77.4% of patients with sepsis in the ICU of a tertiary hospital in China were on mechanical ventilation [18]. One epidemiological study in Beijing showed that the 13.8% of hospitalized patients with sepsis were admitted to the ICU [12]. In the present study, ventilation was only used in 26.6% of patients, most often in elderly patients. Taken together, these data suggest that a large number of patients with sepsis in China are treated in general wards without mechanical ventilation. Critical care physicians may not be sufficiently aware of sepsis, which may lead to inappropriate clinical decision-making and an increase in sepsis-related mortality.

In previous studies, mortality rates for sepsis and severe sepsis in developed countries were 17% and 26%-33.2%, respectively [19, 20]. In low- and middle-income countries, mortality rates for sepsis and severe sepsis were 21.9%-47.3%, and reached 45.6%-52.2% for septic shock [21–25]. In a multicenter study in Asia, in 2009, the ICU mortality rate for severe sepsis was 44.5% [26]. In China, in 2014 and 2015, ICU mortality rates for sepsis and septic shock were 13.1% and 39.0%, respectively, and the hospital mortality rate for sepsis was 33.0% [18]. Similarly, in the present study, the in-hospital mortality rate for sepsis was 31.7%, and increased with age from 4.8% in minors to 58.1% in the elderly. Consistent with these findings, a multicenter study conducted in 2019 in southwest China involving 10,598 children (aged 29 days -18 years) reported the in-hospital mortality rate for severe sepsis and septic shock was 18.8% [27], and a retrospective cohort study of patients with sepsis admitted to public hospitals in Yuetan Subdistrict, Beijing between 2012 and 2014 showed a significant increase in sepsis mortality from 2.4% in patients < 50 years to 30.7% in patients > 90 years [12]. Sepsis mortality depends on factors such as age, race, gender, comorbidities, and degree of organ dysfunction [28]. In China, sepsis mortality is expected to increase with the move towards an aging society and the higher prevalence of comorbidities in elderly individuals.

Globally, mortality associated with sepsis is decreasing by year. Severe sepsis 28-day mortality decreased from 46.9% in 1991-1995 to 29% in 2006-2009 in the US [20], and from 56% in 1993 to 35% in 2001 in France [29]. Absolute mortality in severe sepsis decreased from 35.0% in 2000 to 18.4% in 2012 in ICUs in Australia and New Zealand [30]. Hospital mortality fell from 23.7% in 2008 to 19.7% in 2012 in patients with sepsis in all acute-care hospitals in Catalonia [31]. In contrast, the present study showed an increase in in-hospital mortality in patients with sepsis in China. Long-term epidemiological investigations of sepsis-related mortality in China are limited. However, two multicenter, prospective cohort studies reported that in- hospital mortality in patients with sepsis in China was 33.5% in 2009 [32] and 33.0% in 2014-2015 [18]. The disparate findings between studies may be explained by 1) the use of different definitions of sepsis. Our study used Sepsis-3.0, while the prior studies used Sepsis1.0 or 2.0 [33]; 2) Guidelines for sepsis have evolved since the prior studies were performed [21, 22, 34]; and 3) rates of mechanical ventilation use in sepsis vary, which may influence sepsis-related mortality.

The cost of sepsis may reflect factors such as the Gross Domestic Product (GDP), regional economy, and public opinion of health. In the US, between 1979 and 2000, the cost of sepsis was an estimated $50,000 per patient, resulting in an annual economic burden of $17 billion [35]. Between 2003 and 2007, the economic burden of sepsis in the US increased from $15.4 billion to $24.3 billion [36]. In the UK, in 2002, the cost of sepsis was £25,000 per patient [7]. In contrast, in India, in 2005, the cost of care for sepsis in the ICU was approximately $200 a day [37]. The cost of sepsis in China is not as high as that of developed countries. In 2007, the average ICU cost for sepsis in China was $11,390 per patient, or $502 per patient per day [38]. In our study, median hospitalization cost for all patients was ¥29,453 (15,011, 65,237), and costs increased with age, reaching ¥41,215 (18,991, 88,259) in the elderly. Hospitalization cost showed no significant change from 2012 to 2016, but increased in 2017 and 2018.

## Strengths and limitations

Currently, high-quality epidemiological studies on sepsis in China include data collected over 2-20 months [9, 12, 18, 27, 32]. To the authors’ knowledge, the present study is the longest (7 years) epidemiological investigation of sepsis in secondary and tertiary hospitals in Beijing, using information from 2012-2018 that was derived from the Beijing Public Health System. Information was collated and manually screened to ensure patients with multiple hospitalizations were not overrepresented, which may have biased our findings towards reduced length of hospital stay, in-hospital mortality, and hospitalization cost. The data used in this study were easy and cost-effective to access, and findings provide important information for clinicians and policy makers.

Despite, these strengths, the study was associated with several limitations. First, the data informing this study were acquired from hospitals in Beijing, which may restrict the generalizability of our results across other regions, including those with a lower level of economic development and fewer medical resources. Second, sources of error in retrospective studies include incomplete or missing data. Consequently, the clinical and economic burden of sepsis in China may be more serious than expected; however, our results can be used as a reference for future studies.

## Conclusions

Sepsis is associated with high mortality and cost. From 2012 to 2018, the in-hospital mortality and cost of sepsis in Beijing was higher in older patients and increased by year.

## Data Availability

The datasets generated and analyzed during the present study are available from the corresponding author on reasonable request.
